# Ethyl (2*RS*,3*SR*,4*RS*)-1-ethyl-2-(furan-2-yl)-4-hy­droxy-5-oxopyrrolidine-3-carboxyl­ate

**DOI:** 10.1107/S2414314624010885

**Published:** 2024-11-19

**Authors:** Nor Habibah Mohd Rosli, Mohd Fazli Mohammat, Mohd Abdul Fatah Abdul Manan, David B. Cordes, Aidan P. McKay

**Affiliations:** a Faculty of Applied Sciences Universiti Teknologi MARA (UiTM) Pahang Jengka Campus 26400 Bandar Tun Abdul Razak Jengka Pahang Malaysia; b Centre of Chemical Synthesis & Polymer Technology Institute of Science Universiti Teknologi MARA Puncak Alam 42300 Puncak Alam Selangor Malaysia; c Faculty of Applied Sciences, Universiti Teknologi MARA, 40450 Shah Alam, Selangor, Malaysia; d EaStCHEM School of Chemistry, University of St Andrews, St Andrews, Fife KY16 9ST, United Kingdom; University of Aberdeen, United Kingdom

**Keywords:** crystal structure, oxopyrrolidine, racemic, envelope conformation, hydrogen bonds

## Abstract

The title racemic oxopyrrolidine compound, C_13_H_17_NO_5_, contains three stereogenic centres and crystallizes with two mol­ecules in the asymmetric unit. The five-membered pyrrolidine rings in both mol­ecules exhibit envelope conformations. The *N*-ethyl group of one of the mol­ecules is disordered over two sets of sites in a 0.836 (4):0.164 (4) ratio. In the crystal, both mol­ecules form inversion dimers through pairwise O—H⋯O hydrogen bonds, generating *R*^2^_2_(10) loops, which are linked into a three-dimensional network by weak C—H⋯O hydrogen bonds.

## Abstract 2

The crystal structure of a pyrrolidine analogue obtained from the stereoselective reduction of the enolic form of 4-hy­droxy-2-furyl-pyrrole­carboxyl­ate is described.

## Graphical abstract

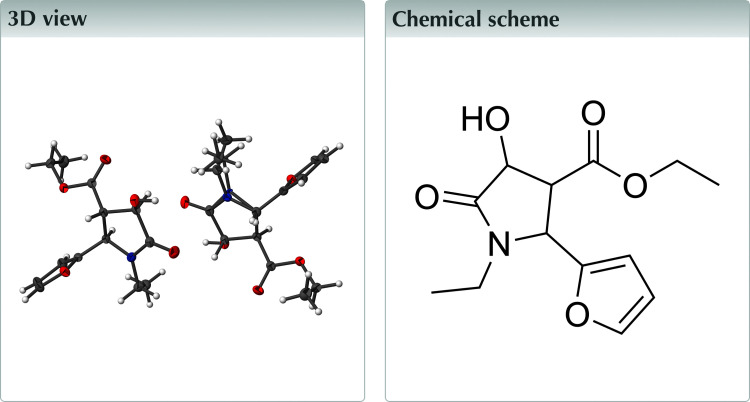

## Structure description

Pyrrolidine compounds have sparked inter­est as a motif in drug candidate mol­ecules due to the non-planar ring structure and stereochemistry that allow for diverse spatial orientations of substituents, which can affect biological functions by altering binding affinity with the target protein (Petri *et al.*, 2021 ▸). The versatility of these scaffolds is highlighted by their occurrences across a range of pharmacological uses, predominantly as anti­virals, anti­diabetics, and anti­cancer agents (Esposito *et al.*, 2020 ▸; Wang *et al.*, 2022 ▸). Hence, various strategies have been employed to obtain stereoselective reactions by exploiting the versatility of the pyrrolidine ring. For instance, a domino reaction of enynal and amino ketone with ZnCl_2_ followed by an aldol reaction yielded functionalized 2-furyl-pyrrolidine derivatives with high diastereoselectivity (Ou *et al.*, 2023 ▸). Herein, we report a facile synthesis approach and crystal structure of the title compound, C_13_H_17_NO_5_, as a continuation of our work to obtain pyrrolidine analogues with potential bioactivity.

**Figure 1:**
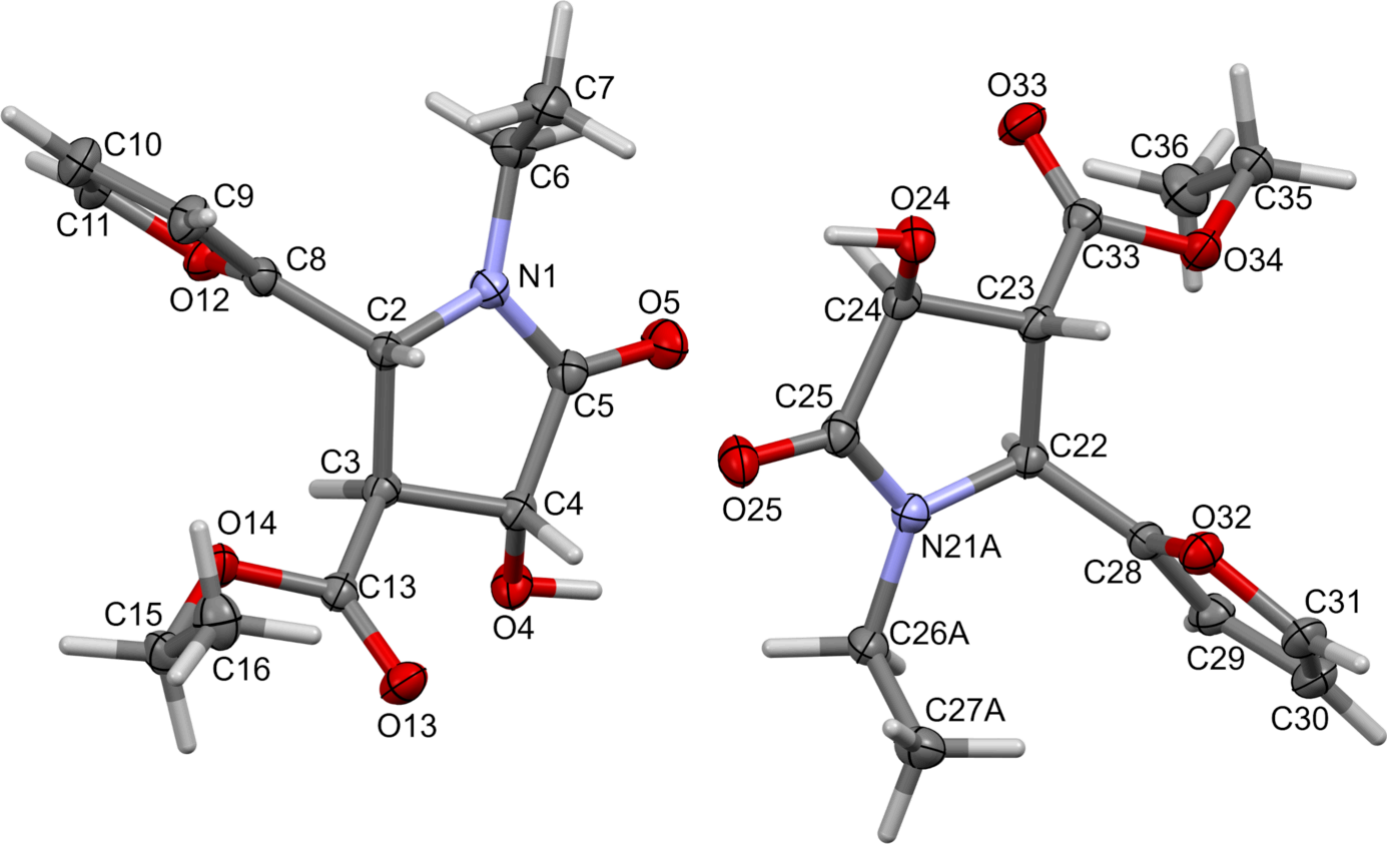
The mol­ecular structure of the title compound, showing displacement ellipsoids drawn at the 50% probability level. Only the major disorder component of the C22 mol­ecule is shown.

The title compound, illustrated in Fig. 1 ▸, crystallizes in the monoclinic space group *P*2_1_/*n* with two independent mol­ecules in the asymmetric unit, each consisting of the five-membered pyrrolidine ring with furan, ethyl ester, and hydroxyl substituents at the 2, 3, and 4 ring positions, respectively. The stereogenic centres of each heterocycle have the relative stereochemistries *RS, SR, RS* at C2, C3, and C4, respectively. The core pyrrolidine rings in both mol­ecules exhibit envelope conformations. Otherwise, the bond lengths and angles in the title compound agree well with similar pyrrolidine containing systems (*e.g*., Abdul Rashid *et al.*, 2023 ▸).

**Figure 2:**
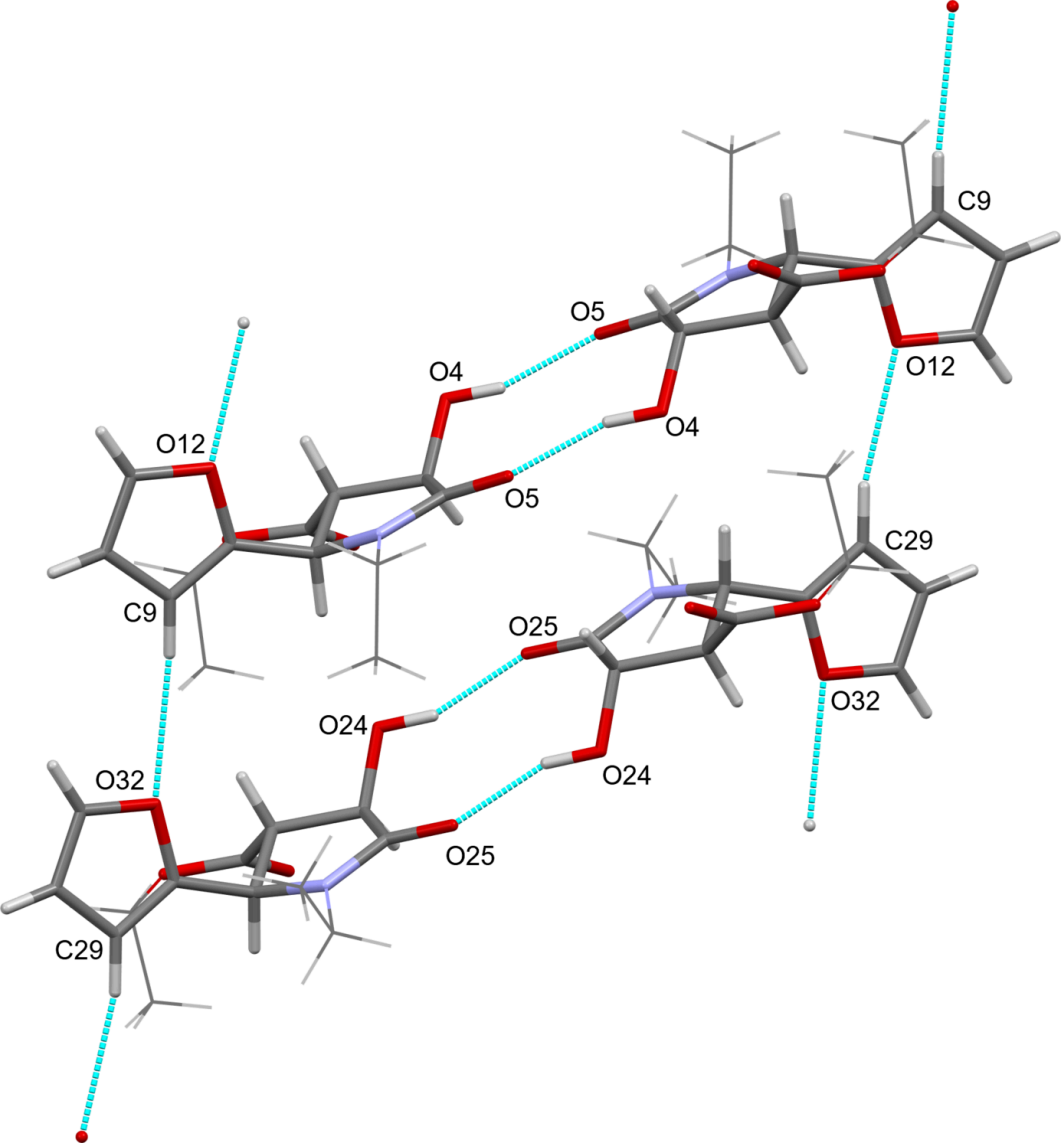
View showing the two independent hydrogen-bonded dimers stacking together through non-classical C—H⋯O hydrogen bonds. The minor component of disorder is omitted and ethyl groups are shown in wireframe representation for clarity.

In the extended structure of the title compound, both independent mol­ecules form reciprocal O—H⋯O hydrogen bonded dimers with inversion-symmetry-related neighbours, in a 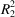
(10) fashion, between the hy­droxy and carbonyl substituents of the pyrrolidine ring. (Table 1 ▸, Fig. 2 ▸). These dimers then stack together, with independent mol­ecules alternating, along [100] through non-classical C—H⋯O hydrogen bonds between adjacent furan moieties, which then further assemble into a three-dimensional network with support from non-classical hydrogen bonds between furan moieties and adjacent ester carbonyl O atoms.

## Synthesis and crystallization

The 2-furyl-pyrrole­carboxyl­ate precursor was synthesized using our published methods for related compounds (Mohammat *et al.*, 2015 ▸) through the multiple-component reaction of sodium diethyl oxalacetate, furfural and ethyl­amine. The title compound was synthesized by adding acetic acid (0.28 ml, 3.83 mmol) followed by sodium borohydride (0.16 g, 4.21 mmol) to a stirred solution of 2-furyl-pyrrole­carboxyl­ate (1.02 g, 3.83 mmol) in 20 ml di­chloro­methane at 0°C. The mixture was allowed to stir for 1 h at 0°C and a further 8 h at room temperature. The solvent was removed in *vacuo* and the crude product was dissolved in ethyl acetate and was extracted with water. The combined organic layers were washed with NaHCO_3_, dried over anhydrous MgSO_4_, and concentrated under reduced pressure. The crude product was subjected to column chromatography using mixed eluents of ethyl acetate: hexane (1:1) (yield: 0.44 g, 43%). m.p. 259–260°C; FT—IR (ATR, ν, cm^−1^) 1733 (C=O), 1695 (C=O); ^1^H NMR (400 MHz, chloro­form-*d_1_*) δ 7.39 (*d*, *J* = 1.9 Hz, 1H, HC=C), 6.38 (*d*, *J* = 3.0 Hz, 1H, HC=C) 6.32 (*dd*, *J* = 3.2, 1.8 Hz, 1H, HC=C), 4.75 (*d*, *J* = 8.5 Hz, 1H, NCH), 4.52 (*d*, *J* = 8.7 Hz, 1H, HCOH), 4.13 (*m*, *J* = 7.8, 6.7 Hz, 2H, OCH_2_), 3.46 (*m*, *J* = 14.6, 7.3 Hz, 1H, NCH_2_), 3.32 (*t, J* = 8.6 Hz, 1H, HCC=O), 2.73 (*m*, *J* = 14.2, 7.2 Hz, 1H, NCH_2_), 1.17 (*t*, *J* = 7.1 Hz, 3H, CH_3_), 0.89 (*t*, *J* = 7.3 Hz, 3H, CH_3_); ^13^C NMR (101 MHz, chloro­form-*d_1_*) δ 171.73 (C=O), 171.08 (C=O), 149.17 (quat. C), 143.84 (HC=C), 110.83 (HC=C), 110.72 (HC=C), 72.29 (HCOH), 61.82 (CH_2_), 54.13 (NCH), 51.93 (HCC=O), 36.19 (NCH_2_), 14.18 (CH_3_), 12.43 (CH_3_); GC–MS: *m*/*z* [*M*^+^] = calculated for C_13_H_17_NO_5_: 267.11; found 267.1. Crystals suitable for X-ray diffraction studies were grown by slow evaporation of an ethyl acetate solution at room temperature.

## Refinement

Crystal data, data collection and structure refinement details are summarized in Table 2 ▸. The NEt group on one mol­ecule (N21—C26—C27) is disordered over two orientations and was modelled with geometric and displacement-factor restraints on the minor component.

## Supplementary Material

Crystal structure: contains datablock(s) I. DOI: 10.1107/S2414314624010885/hb4491sup1.cif

Structure factors: contains datablock(s) I. DOI: 10.1107/S2414314624010885/hb4491Isup2.hkl

Supporting information file. DOI: 10.1107/S2414314624010885/hb4491Isup3.cml

CCDC reference: 2401488

Additional supporting information:  crystallographic information; 3D view; checkCIF report

## Figures and Tables

**Table 1:** Hydrogen-bond geometry (Å, °)

*D*—H⋯*A*	*D*—H	H⋯*A*	*D*⋯*A*	*D*—H⋯*A*
O4—H4⋯O5^i^	0.913 (12)	1.869 (13)	2.7408 (10)	158.9 (14)
O24—H24⋯O25^ii^	0.925 (12)	1.852 (13)	2.7323 (10)	158.1 (14)
C9—H9⋯O32^ii^	0.95	2.53	3.4086 (13)	154
C11—H11⋯O13^iii^	0.95	2.35	3.2758 (13)	165
C29—H29⋯O12^i^	0.95	2.48	3.3717 (13)	156
C31—H31⋯O33^iv^	0.95	2.33	3.2525 (13)	164

Symmetry codes: (i) 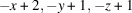; (ii) 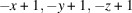; (iii) 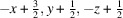; (iv) 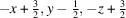.

**Table 2:** Experimental details

Crystal data	
Chemical formula	C_13_H_17_NO_5_
*M*_r_	267.27
Crystal system, space group	Monoclinic, *P*2_1_/*n*
Temperature (K)	100
*a*, *b*, *c* (Å)	11.15335 (7), 17.41068 (9), 14.06240 (9)
β (°)	108.1547 (7)
*V* (Å^3^)	2594.80 (3)
*Z*	8
Radiation type	Cu *K*α
μ (mm^−1^)	0.89
Crystal size (mm)	0.14 × 0.07 × 0.03

Data collection	
Diffractometer	Rigaku XtaLAB P200K
Absorption correction	Multi-scan (*CrysAlis PRO*; Rigaku OD, 2024 ▸)
*T*_min_, *T*_max_	0.790, 1.000
No. of measured, independent and observed [*I* > 2σ(*I*)] reflections	95957, 5344, 4906
*R*_int_	0.069
(sin θ/λ)_max_ (Å^−1^)	0.628

Refinement	
*R*[*F*^2^ > 2σ(*F*^2^)], *wR*(*F*^2^), *S*	0.036, 0.107, 1.08
No. of reflections	5344
No. of parameters	384
No. of restraints	22
H-atom treatment	H atoms treated by a mixture of independent and constrained refinement
Δρ_max_, Δρ_min_ (e Å^−3^)	0.31, −0.20

Computer programs: *CrysAlis PRO* (Rigaku OD, 2024 ▸), *SHELXT2018/2* (Sheldrick, 2015*a* ▸), *SHELXL2019/3* (Sheldrick, 2015*b* ▸), *Mercury* (Macrae *et al.*, 2020 ▸), *OLEX2* (Dolomanov *et al.*, 2009 ▸) and *publCIF* (Westrip, 2010 ▸).

## full crystallographic data

### Ethyl (2RS,3SR,4RS)-1-ethyl-2-(furan-2-yl)-4-hydroxy-5-oxopyrrolidine-3-carboxylate Crystal data

**Table:**

C_13_H_17_NO_5_	*F*(000) = 1136
*M**_r_* = 267.27	*D*_x_ = 1.368 Mg m^−^^3^
Monoclinic, *P*2_1_/*n*	Cu *K*α radiation, λ = 1.54184 Å
*a* = 11.15335 (7) Å	Cell parameters from 50600 reflections
*b* = 17.41068 (9) Å	θ = 4.1–75.1°
*c* = 14.06240 (9) Å	µ = 0.89 mm^−^^1^
β = 108.1547 (7)°	*T* = 100 K
*V* = 2594.80 (3) Å^3^	Prism, colourless
*Z* = 8	0.14 × 0.07 × 0.03 mm

### Ethyl (2RS,3SR,4RS)-1-ethyl-2-(furan-2-yl)-4-hydroxy-5-oxopyrrolidine-3-carboxylate Data collection

**Table:**

Rigaku XtaLAB P200K diffractometer	5344 independent reflections
Radiation source: Rotating Anode, Rigaku MM-007HF	4906 reflections with *I* > 2σ(*I*)
Rigaku Osmic Confocal Optical System monochromator	*R*_int_ = 0.069
Detector resolution: 5.8140 pixels mm^-1^	θ_max_ = 75.5°, θ_min_ = 4.2°
shutterless scans	*h* = −13→13
Absorption correction: multi-scan (CrysAlisPro; Rigaku OD, 2024)	*k* = −21→21
*T*_min_ = 0.790, *T*_max_ = 1.000	*l* = −17→17
95957 measured reflections	

### Ethyl (2RS,3SR,4RS)-1-ethyl-2-(furan-2-yl)-4-hydroxy-5-oxopyrrolidine-3-carboxylate Refinement

**Table:**

Refinement on *F*^2^	Primary atom site location: dual
Least-squares matrix: full	Hydrogen site location: mixed
*R*[*F*^2^ > 2σ(*F*^2^)] = 0.036	H atoms treated by a mixture of independent and constrained refinement
*wR*(*F*^2^) = 0.107	*w* = 1/[σ^2^(*F*_o_^2^) + (0.0599*P*)^2^ + 0.6008*P*] where *P* = (*F*_o_^2^ + 2*F*_c_^2^)/3
*S* = 1.08	(Δ/σ)_max_ = 0.001
5344 reflections	Δρ_max_ = 0.31 e Å^−^^3^
384 parameters	Δρ_min_ = −0.20 e Å^−^^3^
22 restraints	

### Ethyl (2RS,3SR,4RS)-1-ethyl-2-(furan-2-yl)-4-hydroxy-5-oxopyrrolidine-3-carboxylate Special details

**Table:**

Geometry. All esds (except the esd in the dihedral angle between two l.s. planes) are estimated using the full covariance matrix. The cell esds are taken into account individually in the estimation of esds in distances, angles and torsion angles; correlations between esds in cell parameters are only used when they are defined by crystal symmetry. An approximate (isotropic) treatment of cell esds is used for estimating esds involving l.s. planes.
Refinement. The O-bound H atoms were located in a difference map and refined isotropically with a distance restraint. The C-bound H atoms were located geometrically (C—H = 0.95–1.00 Å) and refined as riding atoms. The constraint *U*_iso_(H) = 1.2*U*_eq_(C) or 1.5*U*_eq_(methyl C) was applied in all cases. The NEt group on one molecule (N21—C26—C27) was disordered over two positions and modelled with geometric and thermal restraints on minor component due to instability from low (<20%) occupancy.

### Ethyl (2RS,3SR,4RS)-1-ethyl-2-(furan-2-yl)-4-hydroxy-5-oxopyrrolidine-3-carboxylate Fractional atomic coordinates and isotropic or equivalent isotropic displacement parameters (Å^2^)

**Table:**

	*x*	*y*	*z*	*U*_iso_*/*U*_eq_	Occ. (<1)
O4	0.92054 (7)	0.53494 (4)	0.34391 (6)	0.02196 (18)	
H4	0.9694 (13)	0.5029 (8)	0.3919 (10)	0.036 (4)*	
O5	0.89935 (7)	0.56424 (5)	0.54461 (6)	0.02841 (19)	
O12	0.74321 (7)	0.78349 (4)	0.32650 (6)	0.02110 (17)	
O13	0.62305 (7)	0.50977 (4)	0.17572 (6)	0.02460 (18)	
O14	0.57795 (7)	0.63387 (4)	0.13538 (5)	0.02211 (17)	
O24	0.57281 (7)	0.46110 (4)	0.65771 (6)	0.02178 (17)	
H24	0.5220 (13)	0.4929 (8)	0.6090 (10)	0.037 (4)*	
O25	0.62108 (7)	0.45310 (5)	0.46170 (6)	0.02536 (18)	
O32	0.75626 (7)	0.22738 (4)	0.67378 (6)	0.02102 (17)	
O33	0.86974 (7)	0.49392 (4)	0.83546 (6)	0.02598 (18)	
O34	0.92397 (7)	0.37003 (4)	0.86969 (5)	0.02279 (18)	
N1	0.74582 (8)	0.64686 (5)	0.45417 (6)	0.01942 (19)	
N21A	0.7786 (3)	0.37341 (15)	0.5535 (2)	0.0190 (5)	0.836 (4)
N21B	0.7542 (12)	0.3575 (7)	0.5456 (10)	0.010 (2)	0.164 (4)
C2	0.66128 (9)	0.65746 (6)	0.35129 (7)	0.0165 (2)	
H2	0.578316	0.632617	0.344977	0.020*	
C3	0.73003 (9)	0.61026 (6)	0.28976 (7)	0.0170 (2)	
H3	0.790211	0.644986	0.270707	0.020*	
C4	0.80571 (9)	0.55040 (6)	0.36278 (7)	0.0177 (2)	
H4A	0.755443	0.502061	0.356627	0.021*	
C5	0.82458 (9)	0.58678 (6)	0.46509 (8)	0.0199 (2)	
C6	0.73069 (10)	0.69195 (6)	0.53755 (8)	0.0236 (2)	
H6A	0.742435	0.747037	0.525414	0.028*	
H6B	0.796986	0.676924	0.599909	0.028*	
C7	0.60199 (11)	0.68079 (7)	0.55144 (9)	0.0272 (2)	
H7A	0.536009	0.696876	0.490555	0.041*	
H7B	0.596623	0.711818	0.608110	0.041*	
H7C	0.590373	0.626466	0.564593	0.041*	
C8	0.63972 (9)	0.73994 (6)	0.32359 (7)	0.0171 (2)	
C9	0.53472 (10)	0.78358 (6)	0.29576 (8)	0.0235 (2)	
H9	0.451126	0.767162	0.288571	0.028*	
C10	0.57344 (11)	0.85940 (6)	0.27913 (9)	0.0275 (2)	
H10	0.520635	0.903043	0.258338	0.033*	
C11	0.69883 (11)	0.85672 (6)	0.29865 (8)	0.0241 (2)	
H11	0.750015	0.899272	0.293969	0.029*	
C13	0.63899 (9)	0.57705 (6)	0.19511 (7)	0.0180 (2)	
C15	0.48291 (10)	0.61143 (6)	0.04229 (8)	0.0240 (2)	
H15A	0.515009	0.567984	0.011778	0.029*	
H15B	0.466014	0.654974	−0.005324	0.029*	
C16	0.36226 (11)	0.58807 (7)	0.06123 (9)	0.0299 (3)	
H16A	0.298029	0.576211	−0.002580	0.045*	
H16B	0.332593	0.630269	0.094239	0.045*	
H16C	0.377678	0.542549	0.104343	0.045*	
C22	0.84715 (9)	0.35231 (6)	0.65609 (7)	0.0187 (2)	
H22	0.933196	0.375704	0.673823	0.022*	0.836 (4)
H22A	0.929708	0.377914	0.663058	0.022*	0.164 (4)
C23	0.77142 (9)	0.39493 (6)	0.71602 (7)	0.0175 (2)	
H23	0.712657	0.357492	0.732273	0.021*	
C24	0.69331 (9)	0.45539 (6)	0.64507 (8)	0.0183 (2)	
H24A	0.737082	0.506209	0.658916	0.022*	
C25	0.68947 (10)	0.42710 (6)	0.54179 (8)	0.0206 (2)	
C26A	0.81388 (15)	0.34238 (9)	0.46906 (10)	0.0226 (4)	0.836 (4)
H26A	0.905578	0.331540	0.491361	0.027*	0.836 (4)
H26B	0.797032	0.381674	0.415622	0.027*	0.836 (4)
C27A	0.74323 (14)	0.26966 (10)	0.42640 (11)	0.0280 (4)	0.836 (4)
H27A	0.769527	0.252054	0.369743	0.042*	0.836 (4)
H27B	0.652344	0.280025	0.403650	0.042*	0.836 (4)
H27C	0.762064	0.229808	0.478174	0.042*	0.836 (4)
C26B	0.7672 (7)	0.3099 (5)	0.4613 (5)	0.0171 (16)	0.164 (4)
H26C	0.700974	0.324577	0.398667	0.020*	0.164 (4)
H26D	0.755223	0.255071	0.474579	0.020*	0.164 (4)
C27B	0.8968 (6)	0.3210 (4)	0.4482 (5)	0.0252 (19)	0.164 (4)
H27D	0.901656	0.291392	0.390257	0.038*	0.164 (4)
H27E	0.962137	0.303087	0.508468	0.038*	0.164 (4)
H27F	0.909998	0.375552	0.437640	0.038*	0.164 (4)
C28	0.86200 (9)	0.26813 (6)	0.67484 (7)	0.0180 (2)	
C29	0.96131 (10)	0.22029 (6)	0.68922 (8)	0.0221 (2)	
H29	1.044985	0.234227	0.692887	0.027*	
C30	0.91611 (11)	0.14452 (6)	0.69787 (8)	0.0265 (2)	
H30	0.963761	0.098313	0.708368	0.032*	
C31	0.79324 (11)	0.15164 (6)	0.68823 (8)	0.0251 (2)	
H31	0.739075	0.110162	0.690986	0.030*	
C33	0.85854 (9)	0.42696 (6)	0.81275 (8)	0.0191 (2)	
C35	1.02196 (10)	0.39294 (6)	0.96107 (8)	0.0231 (2)	
H35A	1.047642	0.348019	1.005995	0.028*	
H35B	0.988199	0.432567	0.996345	0.028*	
C36	1.13534 (11)	0.42451 (7)	0.93702 (9)	0.0298 (3)	
H36A	1.111568	0.471708	0.897679	0.045*	
H36B	1.165443	0.386445	0.898403	0.045*	
H36C	1.202567	0.435838	0.999384	0.045*	

### Ethyl (2RS,3SR,4RS)-1-ethyl-2-(furan-2-yl)-4-hydroxy-5-oxopyrrolidine-3-carboxylate Atomic displacement parameters (Å^2^)

**Table:**

	*U*^11^	*U*^22^	*U*^33^	*U*^12^	*U*^13^	*U*^23^
O4	0.0195 (4)	0.0234 (4)	0.0257 (4)	0.0059 (3)	0.0110 (3)	0.0062 (3)
O5	0.0287 (4)	0.0316 (4)	0.0220 (4)	0.0112 (3)	0.0038 (3)	0.0033 (3)
O12	0.0218 (4)	0.0168 (4)	0.0264 (4)	−0.0026 (3)	0.0099 (3)	0.0002 (3)
O13	0.0269 (4)	0.0170 (4)	0.0282 (4)	0.0017 (3)	0.0063 (3)	−0.0030 (3)
O14	0.0263 (4)	0.0180 (4)	0.0188 (4)	−0.0001 (3)	0.0024 (3)	0.0013 (3)
O24	0.0194 (4)	0.0245 (4)	0.0237 (4)	0.0049 (3)	0.0098 (3)	0.0054 (3)
O25	0.0261 (4)	0.0287 (4)	0.0228 (4)	0.0069 (3)	0.0098 (3)	0.0084 (3)
O32	0.0213 (4)	0.0184 (4)	0.0241 (4)	−0.0016 (3)	0.0081 (3)	−0.0003 (3)
O33	0.0294 (4)	0.0167 (4)	0.0289 (4)	0.0024 (3)	0.0048 (3)	−0.0032 (3)
O34	0.0269 (4)	0.0171 (4)	0.0207 (4)	0.0014 (3)	0.0021 (3)	−0.0001 (3)
N1	0.0212 (4)	0.0200 (4)	0.0169 (4)	0.0024 (3)	0.0057 (3)	0.0005 (3)
N21A	0.0234 (12)	0.0158 (11)	0.0194 (9)	−0.0009 (7)	0.0093 (8)	0.0001 (7)
N21B	0.012 (4)	0.008 (4)	0.005 (3)	0.000 (3)	−0.002 (3)	−0.003 (3)
C2	0.0171 (5)	0.0156 (5)	0.0173 (5)	0.0006 (3)	0.0060 (4)	0.0012 (4)
C3	0.0175 (5)	0.0149 (5)	0.0201 (5)	0.0004 (4)	0.0080 (4)	0.0012 (4)
C4	0.0175 (5)	0.0160 (5)	0.0207 (5)	0.0012 (4)	0.0075 (4)	0.0019 (4)
C5	0.0189 (5)	0.0202 (5)	0.0213 (5)	0.0011 (4)	0.0071 (4)	0.0029 (4)
C6	0.0286 (6)	0.0215 (5)	0.0202 (5)	0.0026 (4)	0.0070 (4)	−0.0017 (4)
C7	0.0345 (6)	0.0262 (6)	0.0252 (5)	0.0023 (4)	0.0154 (5)	−0.0002 (4)
C8	0.0192 (5)	0.0167 (5)	0.0166 (4)	−0.0019 (4)	0.0074 (4)	−0.0001 (4)
C9	0.0222 (5)	0.0200 (5)	0.0283 (5)	0.0027 (4)	0.0081 (4)	0.0024 (4)
C10	0.0351 (6)	0.0181 (5)	0.0294 (6)	0.0056 (4)	0.0100 (5)	0.0041 (4)
C11	0.0364 (6)	0.0148 (5)	0.0226 (5)	−0.0034 (4)	0.0113 (4)	0.0006 (4)
C13	0.0184 (5)	0.0179 (5)	0.0198 (5)	0.0013 (4)	0.0090 (4)	0.0012 (4)
C15	0.0276 (6)	0.0257 (5)	0.0167 (5)	−0.0021 (4)	0.0039 (4)	0.0001 (4)
C16	0.0255 (6)	0.0341 (6)	0.0271 (6)	−0.0007 (5)	0.0040 (5)	0.0045 (5)
C22	0.0192 (5)	0.0186 (5)	0.0200 (5)	0.0021 (4)	0.0085 (4)	0.0015 (4)
C23	0.0185 (5)	0.0148 (5)	0.0205 (5)	−0.0001 (4)	0.0081 (4)	0.0004 (4)
C24	0.0177 (5)	0.0160 (5)	0.0222 (5)	0.0008 (4)	0.0078 (4)	0.0025 (4)
C25	0.0218 (5)	0.0197 (5)	0.0230 (5)	0.0008 (4)	0.0109 (4)	0.0047 (4)
C26A	0.0303 (8)	0.0201 (7)	0.0219 (7)	0.0029 (6)	0.0144 (6)	0.0013 (6)
C27A	0.0349 (8)	0.0277 (9)	0.0230 (7)	−0.0010 (6)	0.0110 (6)	−0.0053 (6)
C26B	0.023 (3)	0.010 (3)	0.017 (3)	−0.007 (3)	0.005 (2)	−0.006 (3)
C27B	0.026 (4)	0.029 (4)	0.022 (3)	−0.001 (3)	0.010 (3)	0.003 (3)
C28	0.0199 (5)	0.0187 (5)	0.0160 (4)	−0.0008 (4)	0.0066 (4)	−0.0006 (4)
C29	0.0217 (5)	0.0227 (5)	0.0219 (5)	0.0036 (4)	0.0065 (4)	−0.0018 (4)
C30	0.0351 (6)	0.0182 (5)	0.0257 (5)	0.0060 (4)	0.0087 (5)	−0.0019 (4)
C31	0.0356 (6)	0.0155 (5)	0.0240 (5)	−0.0030 (4)	0.0090 (5)	−0.0023 (4)
C33	0.0195 (5)	0.0172 (5)	0.0219 (5)	0.0014 (4)	0.0085 (4)	0.0010 (4)
C35	0.0258 (5)	0.0225 (5)	0.0185 (5)	0.0004 (4)	0.0035 (4)	−0.0010 (4)
C36	0.0257 (6)	0.0356 (6)	0.0263 (6)	0.0001 (5)	0.0055 (5)	0.0009 (5)

### Ethyl (2RS,3SR,4RS)-1-ethyl-2-(furan-2-yl)-4-hydroxy-5-oxopyrrolidine-3-carboxylate Geometric parameters (Å, º)

**Table:**

O4—H4	0.913 (12)	C10—H10	0.9500
O4—C4	1.4125 (12)	C10—C11	1.3394 (17)
O5—C5	1.2324 (13)	C11—H11	0.9500
O12—C8	1.3709 (12)	C15—H15A	0.9900
O12—C11	1.3796 (13)	C15—H15B	0.9900
O13—C13	1.2033 (13)	C15—C16	1.5068 (15)
O14—C13	1.3384 (12)	C16—H16A	0.9800
O14—C15	1.4578 (12)	C16—H16B	0.9800
O24—H24	0.925 (12)	C16—H16C	0.9800
O24—C24	1.4130 (12)	C22—H22	1.0000
O25—C25	1.2328 (13)	C22—H22A	1.0000
O32—C28	1.3725 (12)	C22—C23	1.5549 (13)
O32—C31	1.3777 (13)	C22—C28	1.4892 (14)
O33—C33	1.2048 (13)	C23—H23	1.0000
O34—C33	1.3387 (12)	C23—C24	1.5234 (13)
O34—C35	1.4594 (12)	C23—C33	1.5107 (14)
N1—C2	1.4718 (12)	C24—H24A	1.0000
N1—C5	1.3437 (13)	C24—C25	1.5215 (14)
N1—C6	1.4639 (13)	C26A—H26A	0.9900
N21A—C22	1.453 (3)	C26A—H26B	0.9900
N21A—C25	1.337 (3)	C26A—C27A	1.513 (2)
N21A—C26A	1.466 (3)	C27A—H27A	0.9800
N21B—C22	1.579 (13)	C27A—H27B	0.9800
N21B—C25	1.403 (13)	C27A—H27C	0.9800
N21B—C26B	1.491 (14)	C26B—H26C	0.9900
C2—H2	1.0000	C26B—H26D	0.9900
C2—C3	1.5574 (13)	C26B—C27B	1.525 (9)
C2—C8	1.4877 (13)	C27B—H27D	0.9800
C3—H3	1.0000	C27B—H27E	0.9800
C3—C4	1.5208 (13)	C27B—H27F	0.9800
C3—C13	1.5148 (14)	C28—C29	1.3497 (14)
C4—H4A	1.0000	C29—H29	0.9500
C4—C5	1.5252 (14)	C29—C30	1.4310 (15)
C6—H6A	0.9900	C30—H30	0.9500
C6—H6B	0.9900	C30—C31	1.3402 (17)
C6—C7	1.5202 (15)	C31—H31	0.9500
C7—H7A	0.9800	C35—H35A	0.9900
C7—H7B	0.9800	C35—H35B	0.9900
C7—H7C	0.9800	C35—C36	1.5107 (15)
C8—C9	1.3477 (14)	C36—H36A	0.9800
C9—H9	0.9500	C36—H36B	0.9800
C9—C10	1.4306 (15)	C36—H36C	0.9800
			
C4—O4—H4	110.1 (9)	N21A—C22—C28	114.80 (13)
C8—O12—C11	106.17 (8)	N21B—C22—H22A	111.9
C13—O14—C15	116.79 (8)	C23—C22—N21B	101.8 (5)
C24—O24—H24	109.6 (9)	C23—C22—H22	108.2
C28—O32—C31	106.05 (8)	C23—C22—H22A	111.9
C33—O34—C35	116.32 (8)	C28—C22—N21B	103.5 (4)
C5—N1—C2	113.94 (8)	C28—C22—H22	108.2
C5—N1—C6	124.09 (9)	C28—C22—H22A	111.9
C6—N1—C2	121.44 (8)	C28—C22—C23	115.02 (8)
C22—N21A—C26A	121.3 (2)	C22—C23—H23	108.6
C25—N21A—C22	116.1 (2)	C24—C23—C22	105.74 (8)
C25—N21A—C26A	122.3 (2)	C24—C23—H23	108.6
C25—N21B—C22	105.0 (7)	C33—C23—C22	111.02 (8)
C25—N21B—C26B	128.8 (11)	C33—C23—H23	108.6
C26B—N21B—C22	123.6 (10)	C33—C23—C24	114.25 (8)
N1—C2—H2	108.9	O24—C24—C23	109.79 (8)
N1—C2—C3	101.80 (7)	O24—C24—H24A	109.8
N1—C2—C8	112.34 (8)	O24—C24—C25	113.44 (8)
C3—C2—H2	108.9	C23—C24—H24A	109.8
C8—C2—H2	108.9	C25—C24—C23	103.99 (8)
C8—C2—C3	115.55 (8)	C25—C24—H24A	109.8
C2—C3—H3	108.5	O25—C25—N21A	126.46 (16)
C4—C3—C2	104.61 (8)	O25—C25—N21B	121.1 (6)
C4—C3—H3	108.5	O25—C25—C24	125.35 (9)
C13—C3—C2	112.23 (8)	N21A—C25—C24	108.04 (16)
C13—C3—H3	108.5	N21B—C25—C24	112.5 (6)
C13—C3—C4	114.26 (8)	N21A—C26A—H26A	109.0
O4—C4—C3	110.41 (8)	N21A—C26A—H26B	109.0
O4—C4—H4A	109.9	N21A—C26A—C27A	113.06 (14)
O4—C4—C5	112.93 (8)	H26A—C26A—H26B	107.8
C3—C4—H4A	109.9	C27A—C26A—H26A	109.0
C3—C4—C5	103.70 (8)	C27A—C26A—H26B	109.0
C5—C4—H4A	109.9	C26A—C27A—H27A	109.5
O5—C5—N1	125.80 (10)	C26A—C27A—H27B	109.5
O5—C5—C4	125.39 (9)	C26A—C27A—H27C	109.5
N1—C5—C4	108.80 (8)	H27A—C27A—H27B	109.5
N1—C6—H6A	109.1	H27A—C27A—H27C	109.5
N1—C6—H6B	109.1	H27B—C27A—H27C	109.5
N1—C6—C7	112.55 (9)	N21B—C26B—H26C	109.5
H6A—C6—H6B	107.8	N21B—C26B—H26D	109.5
C7—C6—H6A	109.1	N21B—C26B—C27B	110.9 (8)
C7—C6—H6B	109.1	H26C—C26B—H26D	108.1
C6—C7—H7A	109.5	C27B—C26B—H26C	109.5
C6—C7—H7B	109.5	C27B—C26B—H26D	109.5
C6—C7—H7C	109.5	C26B—C27B—H27D	109.5
H7A—C7—H7B	109.5	C26B—C27B—H27E	109.5
H7A—C7—H7C	109.5	C26B—C27B—H27F	109.5
H7B—C7—H7C	109.5	H27D—C27B—H27E	109.5
O12—C8—C2	117.40 (8)	H27D—C27B—H27F	109.5
C9—C8—O12	110.13 (9)	H27E—C27B—H27F	109.5
C9—C8—C2	132.46 (9)	O32—C28—C22	117.33 (8)
C8—C9—H9	126.6	C29—C28—O32	110.18 (9)
C8—C9—C10	106.76 (10)	C29—C28—C22	132.42 (9)
C10—C9—H9	126.6	C28—C29—H29	126.7
C9—C10—H10	126.8	C28—C29—C30	106.69 (10)
C11—C10—C9	106.47 (10)	C30—C29—H29	126.7
C11—C10—H10	126.8	C29—C30—H30	126.8
O12—C11—H11	124.8	C31—C30—C29	106.40 (9)
C10—C11—O12	110.47 (9)	C31—C30—H30	126.8
C10—C11—H11	124.8	O32—C31—H31	124.7
O13—C13—O14	124.54 (9)	C30—C31—O32	110.68 (9)
O13—C13—C3	125.59 (9)	C30—C31—H31	124.7
O14—C13—C3	109.87 (8)	O33—C33—O34	124.45 (10)
O14—C15—H15A	109.5	O33—C33—C23	125.48 (9)
O14—C15—H15B	109.5	O34—C33—C23	110.06 (8)
O14—C15—C16	110.68 (9)	O34—C35—H35A	109.5
H15A—C15—H15B	108.1	O34—C35—H35B	109.5
C16—C15—H15A	109.5	O34—C35—C36	110.60 (9)
C16—C15—H15B	109.5	H35A—C35—H35B	108.1
C15—C16—H16A	109.5	C36—C35—H35A	109.5
C15—C16—H16B	109.5	C36—C35—H35B	109.5
C15—C16—H16C	109.5	C35—C36—H36A	109.5
H16A—C16—H16B	109.5	C35—C36—H36B	109.5
H16A—C16—H16C	109.5	C35—C36—H36C	109.5
H16B—C16—H16C	109.5	H36A—C36—H36B	109.5
N21A—C22—H22	108.2	H36A—C36—H36C	109.5
N21A—C22—C23	102.16 (14)	H36B—C36—H36C	109.5
			
O4—C4—C5—O5	−46.93 (14)	C13—C3—C4—O4	91.18 (10)
O4—C4—C5—N1	133.51 (9)	C13—C3—C4—C5	−147.57 (8)
O12—C8—C9—C10	0.49 (12)	C15—O14—C13—O13	2.18 (14)
O24—C24—C25—O25	49.78 (14)	C15—O14—C13—C3	−177.78 (8)
O24—C24—C25—N21A	−134.40 (14)	C22—N21A—C25—O25	179.90 (12)
O24—C24—C25—N21B	−118.3 (5)	C22—N21A—C25—C24	4.1 (2)
O32—C28—C29—C30	−0.01 (12)	C22—N21A—C26A—C27A	91.9 (2)
N1—C2—C3—C4	25.90 (9)	C22—N21B—C25—O25	170.5 (4)
N1—C2—C3—C13	150.32 (8)	C22—N21B—C25—C24	−20.8 (8)
N1—C2—C8—O12	60.01 (11)	C22—N21B—C26B—C27B	−56.6 (12)
N1—C2—C8—C9	−119.12 (12)	C22—C23—C24—O24	141.47 (8)
N21A—C22—C23—C24	−17.32 (13)	C22—C23—C24—C25	19.78 (10)
N21A—C22—C23—C33	−141.75 (12)	C22—C23—C33—O33	118.97 (11)
N21A—C22—C28—O32	−71.41 (16)	C22—C23—C33—O34	−59.94 (10)
N21A—C22—C28—C29	105.14 (18)	C22—C28—C29—C30	−176.75 (11)
N21B—C22—C23—C24	−31.2 (4)	C23—C22—C28—O32	46.74 (12)
N21B—C22—C23—C33	−155.6 (4)	C23—C22—C28—C29	−136.71 (11)
N21B—C22—C28—O32	−63.4 (5)	C23—C24—C25—O25	169.01 (10)
N21B—C22—C28—C29	113.1 (5)	C23—C24—C25—N21A	−15.17 (15)
C2—N1—C5—O5	−176.23 (10)	C23—C24—C25—N21B	0.9 (5)
C2—N1—C5—C4	3.34 (11)	C24—C23—C33—O33	−0.48 (14)
C2—N1—C6—C7	59.57 (12)	C24—C23—C33—O34	−179.40 (8)
C2—C3—C4—O4	−145.71 (8)	C25—N21A—C22—C23	8.5 (2)
C2—C3—C4—C5	−24.46 (10)	C25—N21A—C22—C28	133.66 (16)
C2—C3—C13—O13	−118.21 (11)	C25—N21A—C26A—C27A	−93.8 (2)
C2—C3—C13—O14	61.74 (10)	C25—N21B—C22—C23	31.7 (8)
C2—C8—C9—C10	179.67 (10)	C25—N21B—C22—C28	151.3 (6)
C3—C2—C8—O12	−56.19 (12)	C25—N21B—C26B—C27B	102.6 (11)
C3—C2—C8—C9	124.68 (12)	C26A—N21A—C22—C23	−176.88 (17)
C3—C4—C5—O5	−166.46 (10)	C26A—N21A—C22—C28	−51.7 (2)
C3—C4—C5—N1	13.97 (10)	C26A—N21A—C25—O25	5.3 (3)
C4—C3—C13—O13	0.67 (14)	C26A—N21A—C25—C24	−170.44 (17)
C4—C3—C13—O14	−179.38 (8)	C26B—N21B—C22—C23	−165.0 (9)
C5—N1—C2—C3	−18.63 (10)	C26B—N21B—C22—C28	−45.4 (11)
C5—N1—C2—C8	−142.84 (9)	C26B—N21B—C25—O25	8.4 (14)
C5—N1—C6—C7	−111.54 (11)	C26B—N21B—C25—C24	177.1 (9)
C6—N1—C2—C3	169.42 (9)	C28—O32—C31—C30	−0.07 (12)
C6—N1—C2—C8	45.21 (12)	C28—C22—C23—C24	−142.36 (9)
C6—N1—C5—O5	−4.52 (17)	C28—C22—C23—C33	93.21 (10)
C6—N1—C5—C4	175.04 (9)	C28—C29—C30—C31	−0.03 (12)
C8—O12—C11—C10	0.04 (12)	C29—C30—C31—O32	0.06 (12)
C8—C2—C3—C4	147.93 (8)	C31—O32—C28—C22	177.33 (9)
C8—C2—C3—C13	−87.66 (10)	C31—O32—C28—C29	0.05 (11)
C8—C9—C10—C11	−0.45 (13)	C33—O34—C35—C36	−74.74 (11)
C9—C10—C11—O12	0.25 (13)	C33—C23—C24—O24	−96.14 (10)
C11—O12—C8—C2	−179.65 (8)	C33—C23—C24—C25	142.16 (8)
C11—O12—C8—C9	−0.34 (11)	C35—O34—C33—O33	−5.23 (15)
C13—O14—C15—C16	78.82 (11)	C35—O34—C33—C23	173.70 (8)

### Ethyl (2RS,3SR,4RS)-1-ethyl-2-(furan-2-yl)-4-hydroxy-5-oxopyrrolidine-3-carboxylate Hydrogen-bond geometry (Å, º)

**Table:**

*D*—H···*A*	*D*—H	H···*A*	*D*···*A*	*D*—H···*A*
O4—H4···O5^i^	0.91 (1)	1.87 (1)	2.7408 (10)	159 (1)
O24—H24···O25^ii^	0.93 (1)	1.85 (1)	2.7323 (10)	158 (1)
C9—H9···O32^ii^	0.95	2.53	3.4086 (13)	154
C11—H11···O13^iii^	0.95	2.35	3.2758 (13)	165
C29—H29···O12^i^	0.95	2.48	3.3717 (13)	156
C31—H31···O33^iv^	0.95	2.33	3.2525 (13)	164

Symmetry codes: (i) −*x*+2, −*y*+1, −*z*+1; (ii) −*x*+1, −*y*+1, −*z*+1; (iii) −*x*+3/2, *y*+1/2, −*z*+1/2; (iv) −*x*+3/2, *y*−1/2, −*z*+3/2.
